# Effects of cinnamaldehyde combined with ultrahigh pressure treatment on the flavor of refrigerated *Paralichthys olivaceus* fillets

**DOI:** 10.1039/d0ra01020k

**Published:** 2020-03-27

**Authors:** Yongxia Xu, Yiming Yin, Honglei Zhao, Qiuying Li, Shumin Yi, Xuepeng Li, Jianrong Li

**Affiliations:** College of Food Science and Engineering, Bohai University, National & Local Joint Engineering Research Center of Storage, Processing and Safety Control Technology for Fresh Agricultural and Aquatic Products No. 19, Keji Road Jinzhou 121013 China xuepengli8234@163.com xuyx1009@126.com +86 416 3719190 +86 416 3719190

## Abstract

The combined effects of cinnamaldehyde (CA) and ultrahigh pressure (UP) treatment on the flavor of olive flounder (*Paralichthys olivaceus*) fillets during storage at 4 °C for 20 days were investigated. Changes in total viable count, trimethylamine, ATP-related compounds, free amino acids, TCA-soluble peptides, electronic nose (E-nose) analysis and sensory quality were measured. The results indicated that CA and UP treatment, especially CA combined with UP, significantly reduced undesirable flavor compounds including inosine, hypoxanthine, TMA, and bitter amino acids, and accumulated pleasant flavor compounds such as inosine monophosphate and umami-related amino acids. In addition, the combination of CA and UP was shown to be more effective for retarding protein degradation and microbial growth than CA or UP treatment alone. In accordance with the results of E-nose analysis and sensory evaluation, CA combined with UP treatment had great potential for improving the flavor quality of refrigerated flounder fillets and extending their storage life.

## Abstract 2

The combined effects of cinnamaldehyde (CA) and ultrahigh pressure (UP) treatment on the flavor of olive flounder (*Paralichthys olivaceus*) fillets during storage at 4 °C for 20 days were investigated.

## Graphical abstract

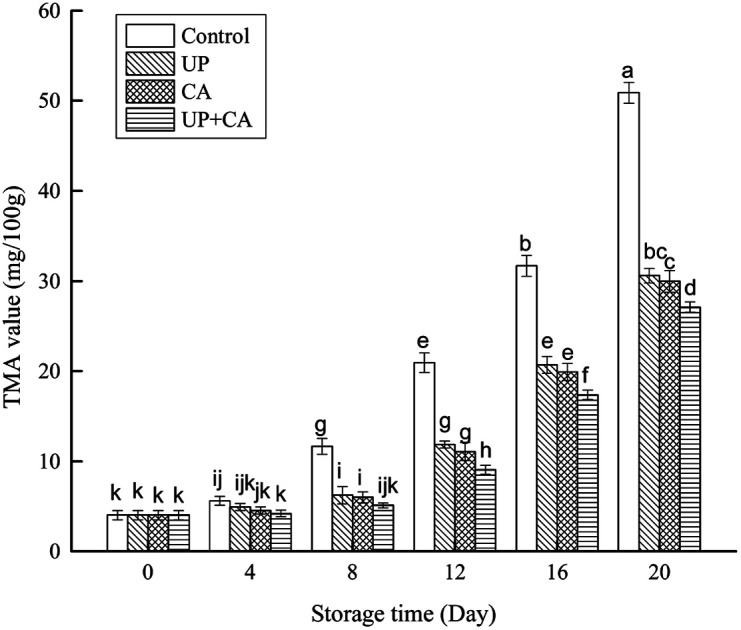

## 1. Introduction

Olive flounder (*Paralichthys olivaceus*), a marine flatfish species, is popular with consumers owing to its pleasant flavor and abundant nutritional value. It is economically important for fisheries and aquaculture, serving as a precious fishing resource in Asia.^1^ Recently, with the development of cold chain logistics, as well as changes in consumption concepts, sales of flounder fillet are growing rapidly. Nevertheless, raw fish spoils easily and develops an unpleasant flavor during post-mortem storage. The spoilage of raw fish is attributed to the actions of endogenous enzymes, microbial enzymes and lipid oxidation, leading to the deterioration of flavor and texture to the point of a loss of edibility. Flavor variation is critical for determining consumers' preferences, often foreshadowing changes in the quality of fish.^2,3^ Therefore, it is essential to study how to prolong the shelf-life and maintain good flavor quality of refrigerated fish fillets.

Recently, cinnamon essential oil (CEO) has been considered a natural food preservative and is widely applied in aquatic products due to its excellent bacteriostatic and antioxidant properties.^4^ Cinnamaldehyde (CA) is the main active component of CEO, accounting for 60–75% of the total oil. It has been identified as GRAS (Generally Recognized as Safe) and can be applied in food or antimicrobial food packaging according to the U.S. Food and Drug Administration.^5,6^ Many researchers have explored the effect of cinnamon essential oil (CEO) on the quality and shelf-life of refrigerated aquatic products such as common carp (*Cyprinus carpio*),^7^ Pacific white shrimp,^8^ and rainbow trout.^9^ These results suggested the positive effectiveness of CEO in prolonging the shelf-life of aquatic products during refrigeration. However, the use of essential oils in fish preservation is limited based on their peculiar flavors and aromas, which affect sensory receptivity.^10^ Lyu *et al.*^11^ found that gamma radiation combined with CEO had a synergistic effect on maintaining fish quality, and additionally, the combination could reduce the radiation dose and concentration of CEO without diminishing the preservation effect. Thus, another preservation method in combination with CA is required to reduce its dosage and organoleptic impact on aquatic products.

Currently, the multi-hurdle technology has been widely used in food preservation. Ultrahigh pressure processing (UPP), as a non-thermal and promising technology, is commonly a feasible hurdle alternative.^12^ UPP only acts on non-covalent bonded structures without damaging the protein's primary structure. It can deactivate spoilage microorganisms and enzymes, prolonging the shelf-life of raw fish and processed products during refrigerated storage.^13^ In particular, ultrahigh pressure (UP) affects cell membranes' permeability *via* liquid medium and disturbs active transport mechanisms, resulting in an absence of nutrients, pH transformations and ultimately cell death.^14,15^ In general, UPP can extend the shelf-life and improve the odor, taste, physicochemical properties as well as overall quality of fish muscles during chilled storage.^16^ On the other hand, UPP beyond 150–200 MPa or higher can result in protein denaturation leading to undesired color changes and cooked-like appearance, and even accelerate lipid oxidation.^17^ Therefore, the suitable selection of UPP parameters especially pressure or in combination with other preservation methods can abate the drawbacks and improve its effectiveness. There have been several prior studies on the use of CA or UP in food.^18,19^ However, they are rarely combined for the preservation and flavor retention of fish or other seafoods. Therefore, the present work is aimed at evaluating the effects of CA combined with UP treatment on the flavor quality of *Paralichthys olivaceus* fillets during refrigerated storage.

## 2. Materials and methods

### 2.1. Sample preparation and treatment

Cinnamaldehyde was purchased from Shenzhen Guoxin Essence Perfume Co. Ltd. (Shenzhen, China). Hydroxypropyl-β-cyclodextrin (HP-β-CD) was purchased by Henan Huarui Biotechnology Co. Ltd. (Henan, China). HP-β-CD solution was prepared by blending HP-β-CD with distilled water and stirring at 55 °C until clear. Cinnamaldehyde was added into the prepared HP-β-CD solution and sonicated at 55 °C with an ultrasonic cleaner (KQ-400KDB, Jiangsu, China) until the color of the mixture became turbid milky white. The final preservative solution of CA consisted of 0.2% cinnamaldehyde (w/v) and 0.4% HP-β-CD (w/v). The concentration of the cinnamaldehyde was selected based on our preliminary study.^20^

Fresh whole flounder (weight: 800 ± 100 g) were purchased from Lin Xi Street Aquaculture Market (Jinzhou, China) and instantly transported to the laboratory, where they were killed by percussive stunning. They were filleted by hand, followed by washing with cold sterile water. Two fillets were obtained from each skin-off dorsal muscle of fish. Afterwards, every fillet was cut into a sample with an average weight and length of 100 ± 8 g and 15 ± 0.2 cm. The fillet samples were then randomly divided into four groups: (1) fillets immersed in deionised water (control); (2) fillets treated with deionised water prior to pressurized at 200 MPa for 10 min (UP); (3) fillets immersed in a preservative solution of cinnamaldehyde (CA); (4) fillets immersed in CA solution and then pressurized at 200 MPa for 10 min (UP + CA). The fillets were dipped into the corresponding solution for 30 min. UP treatments were performed in ultrahigh pressure equipment (HPP.L2-600/0.6, Tianjin, China). All samples were separately packed in air-proof polyethylene bags and stored at 4 ± 1 °C for subsequent quality analysis.

### 2.2. Total viable counts (TVC)

TVC of fish samples was determined using AOAC method.^21^ TVC value was determined by the plate count method. The results were reported as lg CFU (colony forming units) g^−1^.

### 2.3. ATP-related compounds

ATP-related compounds analysis was performed according to the method of Cai *et al.*^22^ Determination of ATP-related compounds was performed using a reverse phase HPLC (Agilent1200; Agilent, CA, USA). Nucleotides, nucleosides, and bases were identified by comparing their retention times with those of commercially obtained standards. The content of each compound was calculated according to the peak areas.

### 2.4. Free amino acids (FAA)

Minced fish sample (2 g) was homogenated with 10 mL of 5% trichloracetic acid solution for 1 min. The homogenate was then centrifuged for 10 min at 7720 rpm. The above extraction process was repeated and the blended supernatants were diluted to 25 mL with distilled water. Then, the extract solution (1 mL) was filtered with a 0.22 μm membrane before being analyzed by an automatic amino acid analyzer (L-8900, Hitachi, Japan). The concentration of free amino acids (mg per 100 g sample) was determined by quantifying with standard amino acids.

### 2.5. TCA-soluble peptide

Three grams of chopped flesh were homogenized with 27 mL trichloroacetic acid (5%, w/v). The samples were kept at 4 °C for 1 h and centrifuged at 5460 rpm for 10 min. The content of TCA-soluble peptides in the supernatant was conducted using the method of Lowry^23^ and expressed as μmol tyrosine per g muscle.

### 2.6. Trimethylamine (TMA)

TMA value determination was carried out by the AOAC method^21^ with minor modification. The fish sample (2 g) was homogenized with 50 mL deionized water and 20 mL of 10% trichloroacetic acid. After ultrasonic treatment in an ice bath for 30 min, the sample was centrifuged at a speed of 10 000 rpm at 4 °C for 6 min. The supernatant was neutralized to pH 4 with 1 M NaOH solution and diluted to 50 mL. Then, 4 mL of the above solution was mixed with 10% formaldehyde (1 mL), toluene (5 mL) and 25% KOH (3 mL) in a colorimetric tube, and heated at 30 °C for 10 min. Then, 3 mL of the mixed solution was dried by 0.2 g anhydrous sodium sulfate and then blended with 3 mL picric acid solution (0.02%). The absorbance of the resulting reagent was recorded at 410 nm against the blank. A standard curve of trimethylamine hydrochloride was prepared and the concentration of TMA was calculated and expressed as mg per 100 g sample.

### 2.7. E-nose analysis

The aroma profiles of fish samples treated by different methods were further determined using a PEN3 E-nose sensor system (Airsense Company, Germany). Two grams of minced muscle were placed into glass beaker and immediately sealed with plastic wrap. The beaker was first incubated at 4 °C for 20 min before injection. Then, the headspace gas was injected into the sensor chamber with a flow speed of 300 mL min^−1^. The data collection time of E-nose detection lasted for 120 s.

### 2.8. Sensory evaluation

Sensory characteristics of fillets were assessed according to the method of Zhou, Chong, Ding, Gu, and Liu^24^ with some modifications. Nine panelists graded for six odor attributes (pleasant odor, grassy odor, fishy odor, amine odor, and rancid odor), using a nine-point hedonic scale (1-dislike extremely to 9-like extremely). A sensory score of 4 was deemed as the boundary of acceptability.

### 2.9. Statistical analysis

The mean data of three parallel experiments was the final consequence. All data were performed by one-way-analysis of variance (ANOVA). Means separations were adopted by Duncan test at a significance level of 5%. Principal component analysis (PCA) was applied to analyze E-nose data. Analyses were performed with the software SPSS version 19.0.

## 3. Results and discussion

### 3.1. Changes in TVC

**Fig. 1:**
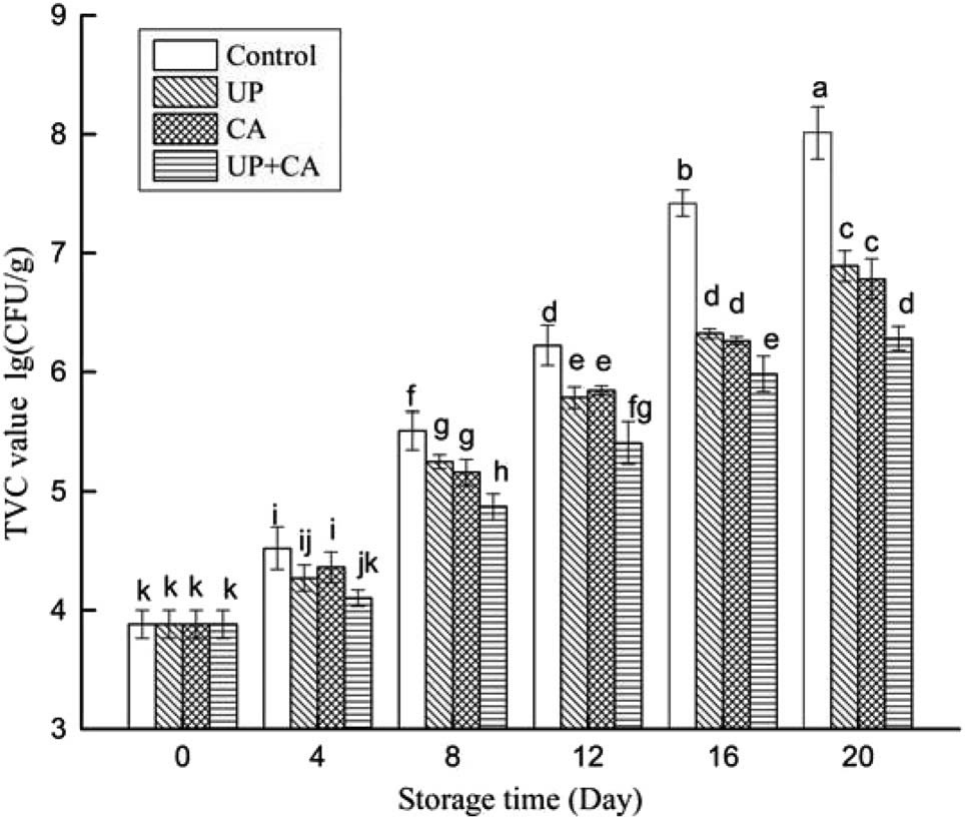
Changes in TVC of flounder fillets during refrigerated storage. Different letters indicate significant differences (*P* < 0.05).

The activity of microorganisms is the main factor responsible for fish spoilage and eventual change of flavor.^25^ Enzymes produced by microbial metabolism could cause protein and lipid degradation, resulting in the generation of volatile products. In general, fish muscles are rich in trimethylamine oxide (TMAO) and free amino acids that can easily form TMA and nitrogenous compounds due to microbial activity, leading to consumers' rejection.^16^ As depicted in Fig. 1, the initial TVC value was 3.88 lg CFU g^−1^ at the first day of storage, indicating that the flounder fillets were of good quality. The TVC values of all the groups increased significantly with the extension of storage time (*P* < 0.05). Additionally, during the entire storage period, the growth rate of treated samples was notably lower than that of the control group (*P* < 0.05), and there was no significant difference in the aerobic bacterial count between the UP and CA groups (*P* > 0.05). After 16 days of storage, the TVC of control samples reached 7.42 lg CFU g^−1^, exceeding the maximum limit (7 lg CFU g^−1^), as provided by ICMSF^26^ for fresh fillets. However, the TVC values of UP- and CA-treated samples reached 6.32 and 6.26 lg CFU g^−1^ on day 16, respectively, and the UP + CA samples showed the slowest growth rate of TVC, reaching 6.28 lg CFU g^−1^ on day 20. CA, as an electro-negative compound, could disturb biological processes involving electron transfer and protein synthesis, thus inhibiting microbial growth.^27^ For ultrahigh pressure processing, it could effectively decrease the initial microbial load in fish muscles as well as the growth of spoilage microorganisms.^16^ Similarly, Ojagh, Núñez-Flores, López-Caballero, Montero, and Gómez-Guillén^17^ also found that the aerobic bacterial count of trout reduced by 5 lg CFU g^−1^ after treatment with 300 MPa for 10 min. In fish, the development of spoilage bacteria such as *pseudomonas* and *S. putrefaciens* can lead to degradation and formation of foul odor during storage.^28^ The results indicated that treating flounder fillet samples with CA combined with UP retarded the microorganic growth synergistically, thus improving the flavor quality and prolonging storage life.

### 3.2. ATP-related compounds analysis

**Fig. 2:**
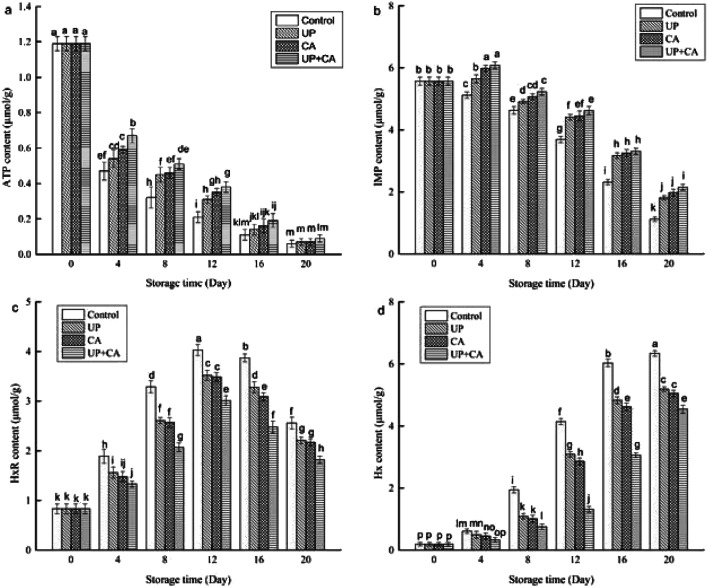
Changes in ATP-related compounds ((a) ATP; (b) IMP; (c) HxR; (d) Hx) of flounder fillets during refrigerated storage.

The concentrations of ATP and its breakdown products are closely related to the flavor and freshness of fish. After death, ATP in fish rapidly breaks down into ADP, AMP and IMP, due to endogenous enzymes. Subsequently, IMP degrades to inosine (HxR) and hypoxanthine (Hx). Among these compounds, IMP plays an important role in desirable flavor, while HxR and Hx are responsible for off-flavor and bitterness in fish muscle.^29^ Hx can be generated by nucleotides' autolytic breakdown or/and bacteria such as *Pseudomonas* spp. and *S. putrefaciens*.^30^ As can be seen in Fig. 2a, the concentration of ATP in the samples had an initial value of 1.19 μmol g^−1^. With the extension of storage time, the concentrations of ATP were found to decrease significantly in all groups (*P* < 0.05), especially during the first 4 days of storage. In addition, the reduction in the ATP content of treated samples was prominently lower than that of the control group. Nevertheless, at the end of the storage period, the ATP content among different groups showed no significant difference (*P* > 0.05), and the ATP content of UP + CA samples decreased by 1.10 μmol g^−1^, which was slower than the other groups. The rapid degradation of ATP in flounder fillets during storage might be caused by the activation of ATP enzymes.^31^ The results of this study suggested that CA and/or UP treatment could affect the activity of ATP enzyme. Tariq *et al.*^32^ reported that CA could inhibit ATPase enzymes and destroy the outer cell membrane. Truong *et al.*^16^ indicated that the activity of Ca-ATPase and Mg-ATPase in sardine was declined with the increase of high pressure treatment from 100 to 500 MPa.

As one of the predominant umami nucleotides, IMP is mainly derived from the decomposition of AMP, due to the presence of AMP-deaminase and acid phosphatase.^29^ The high content of IMP is a delicious flavor enhancer of fish muscle. As shown in Fig. 2b, the IMP content of fresh flounder fillets reached as much as 5.57 μmol g^−1^ at the beginning of storage. Significant decreases in IMP content were observed in control samples throughout the storage time (*P* < 0.05). In the treated samples, the IMP content showed a growing trend in the first 4 days of storage, while significant decreases in IMP content occurred during subsequent storage (*P* < 0.05). Additionally, the IMP concentrations in the treatment groups were prominently higher than those in the control group during the same storage period. After 20 days in storage, the IMP content of fillets treated with UP, CA and UP + CA was 1.81, 1.98 and 2.15 μmol g^−1^, respectively, whereas the control sample reached a concentration of 1.12 μmol g^−1^, indicating that both UP and CA could substantially inhibit the interrelated enzyme activities and further suppress the IMP breakdown.

As presented in Fig. 2c and d, the initial content of Hx was obviously lower than that of HxR. This was in agreement with the results of previous reports on grass carp.^3^ With the extension of storage time, the HxR content increased dramatically (*P* < 0.05) to the highest value on the 12th day and then declined significantly with subsequent storage time (*P* < 0.05). Furthermore, the HxR content of treated samples was notably lower than that of control. The increase could be attributed to IMP consumption by 5′-nucleotidase and the decrease might be due to Hx formation decomposed by HxR. Meanwhile, microbial reproduction might also be a reason for the decline of HxR. Hx is a contributor to off-flavor, and its accumulation is the predominant factor in fish decomposition and poor quality.^33^ During the first 4 days of storage, the Hx content among different groups showed no obvious difference (*P* > 0.05), possibly due to low levels of microorganisms and HxR in the initial storage. However, the Hx content increased sharply after 8 days of storage (*P* < 0.05), reaching 6.34, 5.19, 5.05 and 4.54 μmol g^−1^ when stored for 20 d in control, UP, CA and UP + CA samples, respectively. The Hx content was dramatically lower (*P* < 0.05) in the UP + CA sample, suggesting that UP combined with CA was more effective in restraining microbial growth and protease activity, and thus the fillets maintained better flavor quality throughout the storage.

### 3.3. Free amino acids (FAA) analysis

Table 1 shows the contents of FAA in refrigerated fillets on days 0, 4, 12 and 20. Sixteen amino acids were detected in fresh flounder fillets, and the most abundant FAA was Lys, followed by Ala and Glu, adding up to 66.01% of total FAAs. The FAAs contribute to the taste of fish fillets, specifically umami, sweetness and bitterness. Glu, Asp, Ala, and Gly play an important role in the umami taste of food,^34^ and their total content reached 21.66 mg/100 g in flounder fillets. As shown in Table 1, the total FAA content, as well as that of Gly, Lys, Ser and Met, was found to significantly decrease in all the groups firstly and then increase during the following storage period (*P* < 0.05). At the end of the storage period, the Lys, His, Leu and Phe content presenting for bitterness was lower in the UP + CA group than the other treated groups, while the umami FAAs such as Asp, Glu and Ala had accumulated. The FAAs can be generated by fish muscle proteolysis caused by endogenous and microbial enzymes. The changes in FAAs with storage time depend on the balance between their production and degradation into volatile and nonvolatile compounds.^35^ The decrease in the FAA content in fish muscle during storage might indicate their degradation and metabolism by bacteria,^36^ while the increase might be owing to protein decomposition in fish muscle when subjected to higher proteolytic enzymatic activity after 12 days of storage.^18^ In addition, some FAA concentrations of the UP group were markedly higher than those of the control and CA treatment groups (*P* < 0.05). The results indicated that UP could promote amino acids accumulation, in accordance with the study by Yue *et al.*^36^ who found that high pressure at 200 MPa could enhance the levels of taste FAAs in squid muscles during storage. By contrast with other treated samples, the CA samples had the lowest FAA concentration of 102.62 mg/100 g after 20 days of storage (*P* < 0.05). The CA could effectively inhibit microbial growth and correlative enzymes,^37^ thereby deferring the proteolysis caused by microbial enzymes in the later storage period. Therefore, UP combined with CA treatment might promote the accumulation of umami amino acids and delay the release of bitter amino acids, thus better maintaining the flavor quality of fillets. Similar results were reported by Yu *et al.*^3^ who also found chitosan coating combined with essential oil contributed to the significant accumulation of partial umami-associated FAA and the reduction of off-tasting histidine in refrigerated fillets.

Changes in FAA content of flounder fillets during refrigerated storage^a^

**Table:** 

Storage time	Groups	FAAs content (mg/100 g)								
		Gly	Tyr	Lys	Glu	Met	Phe	Thr	pro	Val
Day 0		2.15 ± 0.07d	1.51 ± 0.04k	32.66 ± 1.44a	5.69 ± 0.18de	1.64 ± 0.08e	1.63 ± 0.06g	2.99 ± 0.18e	0.00 ± 0.00g	2.60 ± 0.06f
Day 4	Control	1.74 ± 0.04gh	1.65 ± 0.06j	9.73 ± 0.23g	6.21 ± 0.28d	0.80 ± 0.06h	1.73 ± 0.03 fg	2.38 ± 0.29e	1.31 ± 0.06f	3.25 ± 0.33cdef
	UP	1.95 ± 0.03e	4.24 ± 0.03d	16.64 ± 1.27f	6.13 ± 0.21de	1.18 ± 0.06g	2.29 ± 0.11e	4.34 ± 0.38d	4.95 ± 0.68b	3.25 ± 0.39cdef
	CA	1.56 ± 0.06h	1.31 ± 0.04l	15.84 ± 0.57f	4.14 ± 0.30f	0.41 ± 0.04i	1.96 ± 0.12f	2.59 ± 0.29e	2.30 ± 0.23e	3.31 ± 0.19cdef
	UP + CA	1.36 ± 0.07i	2.26 ± 0.05i	18.25 ± 1.03ef	3.50 ± 0.23g	1.38 ± 0.04f	1.64 ± 0.08g	2.89 ± 0.36e	2.04 ± 0.30ef	3.01 ± 0.19ef
Day 12	Control	2.53 ± 0.11c	2.64 ± 0.03h	16.40 ± 1.65f	4.08 ± 0.15f	1.59 ± 0.07e	2.65 ± 0.06d	2.89 ± 0.15e	3.18 ± 0.28d	4.71 ± 0.61bcd
	UP	2.33 ± 0.14d	5.03 ± 0.05a	23.13 ± 1.30bcd	6.80 ± 0.20c	1.76 ± 0.04e	3.40 ± 0.21c	5.26 ± 0.39c	4.39 ± 0.47b	4.34 ± 0.59cde
	CA	1.93 ± 0.03ef	4.80 ± 0.07b	16.81 ± 1.37f	5.64 ± 0.25e	0.83 ± 0.06h	1.69 ± 0.08 fg	2.79 ± 0.35e	3.39 ± 0.49 cd	2.30 ± 0.17f
	UP + CA	2.53 ± 0.08c	4.53 ± 0.06c	24.31 ± 0.61bc	6.20 ± 0.18d	2.11 ± 0.06d	3.45 ± 0.16c	5.31 ± 0.61c	4.32 ± 0.41b	4.78 ± 0.71bc
Day 20	Control	8.10 ± 0.20a	3.61 ± 0.04f	26.18 ± 1.48b	9.41 ± 0.25a	2.73 ± 0.07c	5.5 ± 0.25a	5.06 ± 0.42 cd	4.15 ± 0.43bc	9.10 ± 1.44a
	UP	3.83 ± 0.06b	3.80 ± 0.08e	21.23 ± 2.61cde	4.41 ± 0.26f	4.15 ± 0.18a	5.61 ± 0.24a	6.28 ± 0.54 ab	5.84 ± 0.55a	6.16 ± 0.67b
	CA	1.76 ± 0.04 fg	3.39 ± 0.06g	20.80 ± 1.65de	4.05 ± 0.27f	1.19 ± 0.06g	4.33 ± 0.09b	5.53 ± 0.26bc	4.66 ± 0.37b	3.14 ± 0.49def
	UP + CA	3.73 ± 0.02b	4.83 ± 0.05b	20.25 ± 1.39de	7.65 ± 0.23b	3.60 ± 0.07b	4.16 ± 0.07b	6.88 ± 0.83a	6.60 ± 0.83a	8.58 ± 1.68a

a Different lower case letters in different groups from different days indicate a significant difference (*P* < 0.05).

**Table:** 

Storage time	Groups	FAAs content (mg/100 g)							
		His	Arg	Ile	Asp	Leu	Ser	Ala	Total
Day 0		5.80 ± 0.06g	4.70 ± 0.23a	1.53 ± 0.13e	0.45 ± 0.06d	1.65 ± 0.11d	4.18 ± 0.28d	13.38 ± 3.20cde	82.56 ± 6.05d
Day 4	Control	5.77 ± 0.06g	1.95 ± 0.13d	1.46 ± 0.19e	0.35 ± 0.04def	1.79 ± 0.15d	3.98 ± 0.30d	14.45 ± 3.16cde	58.52 ± 5.31e
	UP	5.34 ± 0.16g	3.15 ± 0.18c	1.76 ± 0.30e	0.39 ± 0.08de	2.06 ± 0.40d	4.48 ± 0.28d	16.28 ± 1.48 cd	78.40 ± 5.19d
	CA	5.17 ± 0.20g	3.79 ± 0.17b	1.66 ± 0.13e	0.44 ± 0.01d	1.45 ± 0.33d	2.51 ± 0.18f	9.78 ± 1.32e	58.18 ± 3.57e
	UP + CA	4.98 ± 0.33g	3.34 ± 0.27c	1.51 ± 0.18e	0.46 ± 0.06d	1.81 ± 0.37d	3.24 ± 0.17e	10.69 ± 1.50de	62.33 ± 3.68e
Day 12	Control	9.69 ± 0.17d	0.06 ± 0.04f	2.38 ± 0.27 cd	0.30 ± 0.08ef	3.21 ± 0.30c	5.64 ± 0.32c	18.18 ± 1.53c	80.11 ± 5.52d
	UP	8.99 ± 0.29de	4.28 ± 0.21a	2.60 ± 0.17c	0.70 ± 0.04c	3.31 ± 0.49c	5.53 ± 0.57c	16.98 ± 3.13 cd	98.80 ± 8.30c
	CA	8.56 ± 0.16e	1.85 ± 0.23d	1.84 ± 0.18de	0.74 ± 0.06c	3.65 ± 0.24c	3.99 ± 0.31d	15.68 ± 1.15cde	76.46 ± 3.75d
	UP + CA	7.54 ± 0.31f	4.30 ± 0.27a	2.94 ± 0.25c	1.54 ± 0.03a	4.11 ± 0.31c	4.35 ± 0.25d	18.16 ± 2.06c	100.46 ± 4.31c
Day 20	Control	18.41 ± 0.74a	0.08 ± 0.06f	4.58 ± 0.42a	0.39 ± 0.06de	8.19 ± 0.47a	7.46 ± 0.52a	35.65 ± 4.31a	148.58 ± 0.52a
	UP	13.82 ± 0.29b	0.04 ± 0.01f	4.00 ± 0.44b	0.24 ± 0.03f	7.85 ± 0.87a	6.44 ± 0.41b	24.36 ± 1.26b	118.03 ± 8.29b
	CA	13.12 ± 0.32b	0.39 ± 0.09f	3.98 ± 0.33b	0.65 ± 0.10c	6.23 ± 0.47b	5.19 ± 0.35c	24.26 ± 1.24b	102.62 ± 4.91c
	UP + CA	11.78 ± 0.84c	1.11 ± 0.16e	4.98 ± 0.48a	1.25 ± 0.06b	5.99 ± 0.76b	6.83 ± 0.26 ab	28.88 ± 3.83b	127.08 ± 2.09b

### 3.4. TCA-soluble peptides

**Fig. 3:**
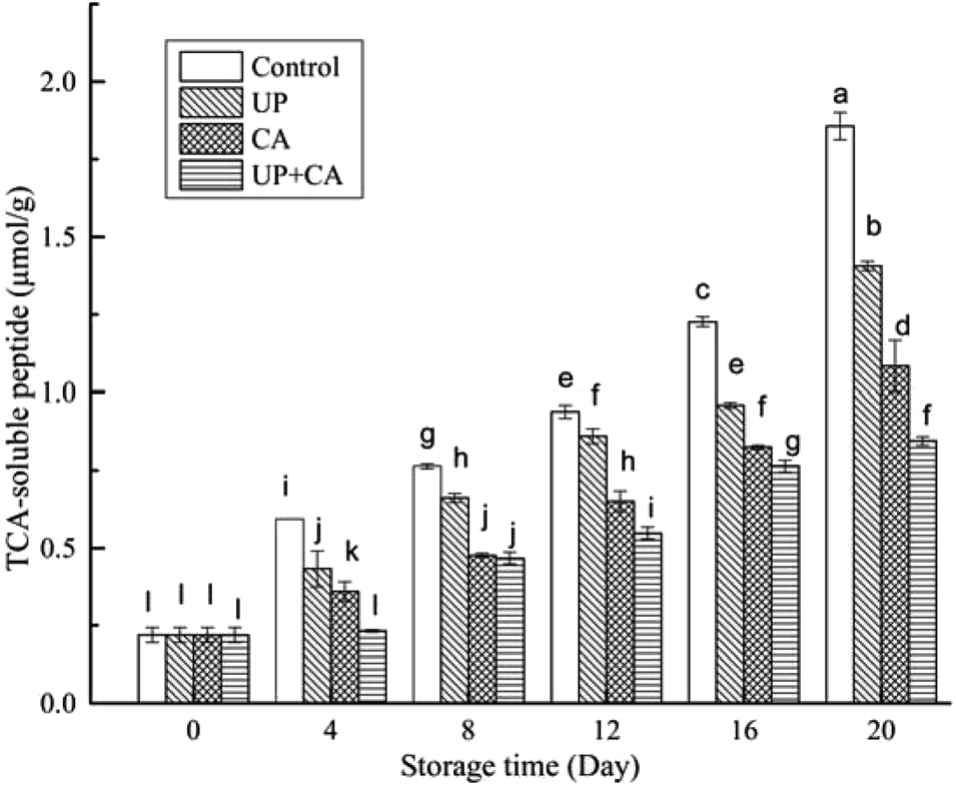
Changes in TCA-soluble peptide of flounder fillets during refrigerated storage. Different letters indicate significant differences (*P* < 0.05).

The proteins in fish flesh are susceptible to decomposition by microorganisms and enzyme, which affects the taste and flavor, as well as the general freshness of fish.^25^ Soluble peptides will be decomposed into amino acids and be further degraded to produce such volatile products as ammonia and amines, aldehydes, thiols, H_2_S, and indole, which finally causes some unpleasant odors.^3^ As shown in Fig. 3, the level of TCA-soluble peptide in the fresh sample was approximately 0.22 μmol g^−1^. The initial TCA-soluble peptides in post-slaughter fillets might be produced by endogenous peptides and the accumulation of their degradation products.^38^ The TCA-soluble peptide content of all the groups increased gradually throughout the storage period (*P* < 0.05), mainly correlating with the activity of autolysis and exogenous proteases.^39^ Besides, the TCA-soluble peptide content in the control group was significantly higher than those in the UP, CA and UP + CA groups at the same storage time (*P* < 0.05), demonstrating the effective inhibition of proteolysis by UP and/or CA treatments. This was consistent with the higher TVC and TMA values of the control sample compared with other treated samples. This result indicated that the control sample might have higher protease activity, leading to an increase in nitrogenous degradation products. In addition, protein catabolism and its nitrogenous degradation products were beneficial for bacteria proliferation, which could further accelerate the decomposition of fish muscle. Therefore, the TCA-soluble peptides still showed a remarkable increase during the later storage period (*P* < 0.05). After 20 days of storage, the TCA-soluble peptide concentration of the control group reached 1.86 μmol g^−1^, while that of the UP, CA and UP + CA treated groups were 1.41, 1.08 and 0.85 μmol g^−1^, respectively. UP could deactivate the autolytic enzymes and microorganisms due to denaturation and/or modification of proteins, resulting in the inhibition of proteolytic degradation in fish muscle. The lower TCA-soluble peptide content of the CA group by comparison with the UP group could be due to superior antioxidative and antibacterial activities of CA. Apparently, the UP + CA group had the lowest TCA-soluble peptide content during storage, indicating that UP combined with CA treatment caused an intensively synergistic effect and better restrained the proteolytic degradation than UP or CA treatment alone.

### 3.5. Changes in TMA content

**Fig. 4:**
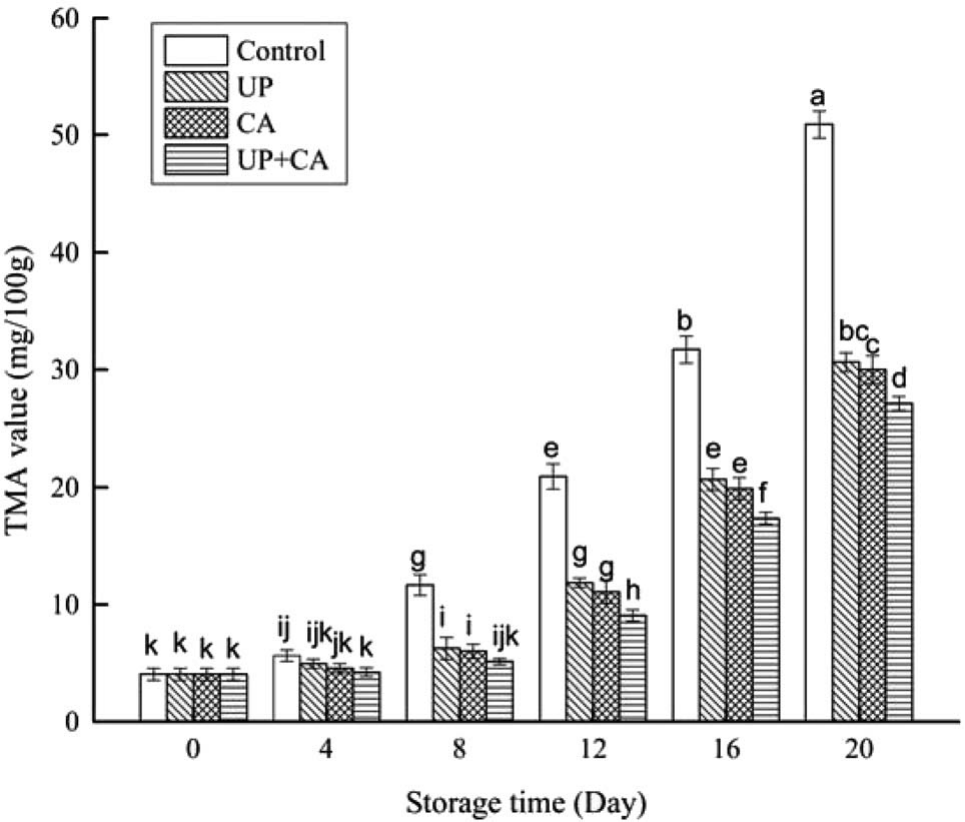
Changes in TMA content of flounder fillets during refrigerated storage. Different letters indicate significant differences (*P* < 0.05).

TMA is one of the main substances accountable for unpleasant fishy odor in aquatic products, which is the major decomposition product of trimethylamine oxide after microbial metabolism.^40^ As presented in Fig. 4, the initial TMA content was 4.01 mg/100 g in fresh sample. The TMA values in the control sample increased significantly as the storage time increased (*P* < 0.05), while the treated samples showed a rapid increase after 8 days of storage (*P* < 0.05). Although the TMA values increased as the storage period progressed in all samples, the values in the treated samples were prominently lower than that of the control, and the differences became remarkable after the eighth day (*P* < 0.05). Besides, there was no obvious difference between the TMA values of the UP- and CA-treated samples at the corresponding storage time (*P* > 0.05). In particular, the control sample increased to the value of 11.64 mg/100 g on the eighth day of storage. From the viewpoint of Özogul *et al.*,^41^ a level of around 10–15 mg TMA/100 g in fish muscle means it is spoiled and unfit for human consumption. On day 12 of storage, the TMA values of UP and CA samples reached 11.83 and 11.07 mg/100 g, respectively. However, the value in the UP + CA samples was still below 10 mg/100 g. Apparently, the accumulation of TMA was inhibited by CA and UP treatments, and the inhibiting effect was effectively promoted by the combination of CA and UP. The lower TMA values in the samples treated with UP may be attributed to the inhibition of proteolytic activity.^42^ In early work, Bindu *et al.*^43^ found that the TMA values of Indian white prawn after high pressure treatment were significantly reduced during chill storage, in accordance with the present results. Additionally, the TMA values measured in this study were observably low in the samples with CA treatments, suggesting that the growth of TMA-producing bacteria such as *Pseudomonas* spp. and *S. putrefaciens* was substantially restrained.^37^

### 3.6. E-nose analysis by PCA

**Fig. 5:**
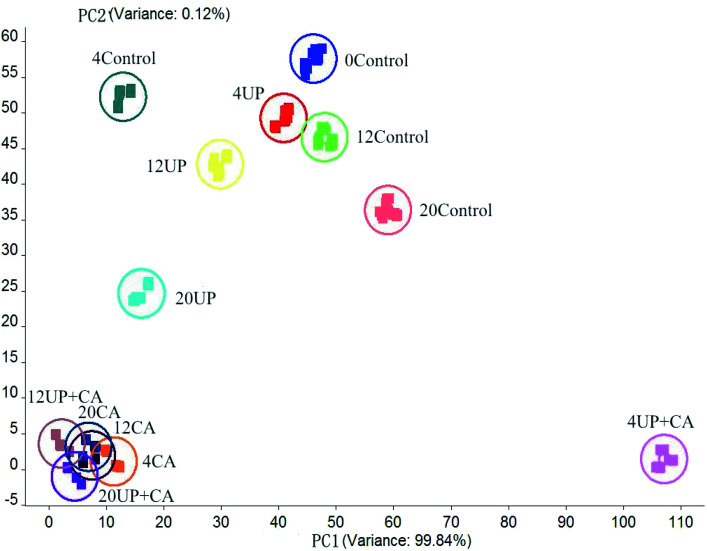
PCA plots of E-nose data for flounder fillets during refrigerated storage.

E-nose technology, as a mimic sense of smell to detect and distinguish odors, is widely applied to assess food quality.^44^ E-nose analysis was carried out to distinguish the aroma profiles of fillets on the 0th, 4th, 12th and 20th days; a PCA loading plot of the different variables of flounder fillet is presented in Fig. 5. The plot was composed of two axes, PC1 and PC2, where PC1 explained 99.84% of the sample variance and PC2 explained just 0.12%. The summed sample variance of PC1 and PC2 was 99.96%. Therefore, the main variations for the volatile components captured by PC1 could help to effectively distinguish flounder fillets with different treatments.^24^ Although PC2 exhibited small variations, it still played an important role in determining certain factors involving the effects of CA treatments. In general, compared with the dots of fresh sample on day 0, the dots corresponding to other samples were distributed to the negative along the PC2 axis. Considering the distribution characteristics of different samples, the points of UP-treated samples were relatively close to those of the control sample, suggesting that the UP treatment made no large differences in the odor composition of the fillets. Moreover, the points of UP and control samples moved gradually farther away from those homologous dots of fresh fish sample during storage. The dots representing the samples treated with CA were all located in a cluster and farther away from other samples, indicating that the effect of CA treatment on the smell distribution of the fillets was apparent. Because CA has a strong odor, it could cover the original fishy aroma and had a powerful influence on the smell of fish samples. By contrast, there were only slight differences existed between the CA treated samples though their dots showed a wide range, because the variance (0.12%) explained by the PC2 axis was extremely minor.

### 3.7. Sensory evaluation

**Fig. 6:**
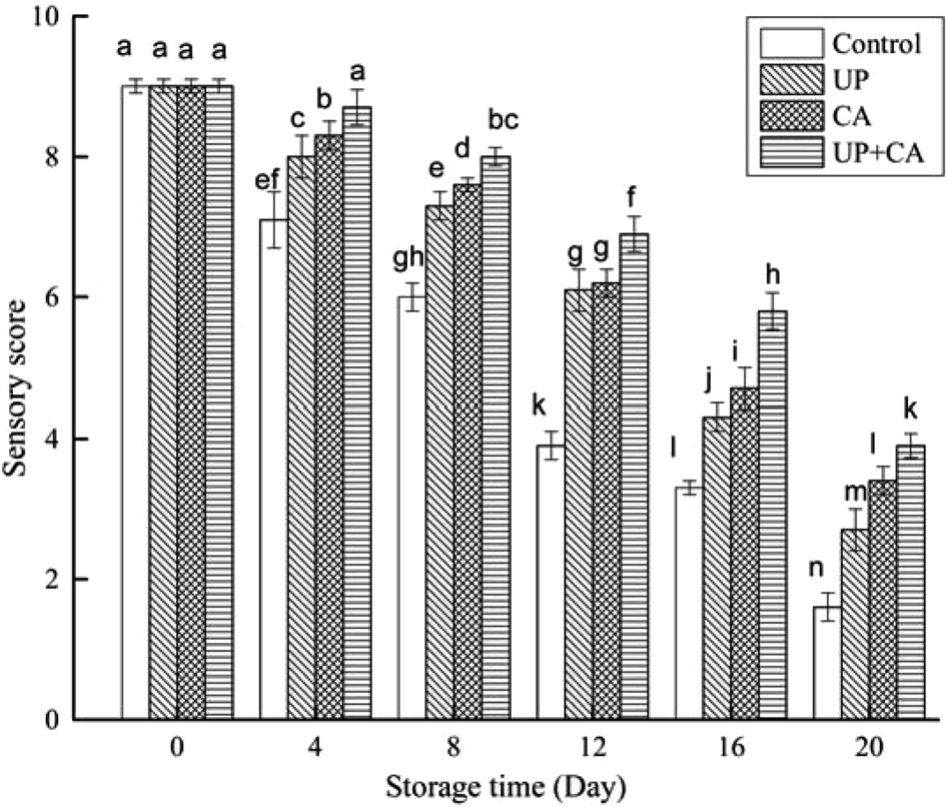
Changes in sensory score of flounder fillets during refrigerated storage. Different letters indicate significant differences (*P* < 0.05).

Cinnamaldehyde itself has some odors and it can alter the flavor of fish when added in large amounts. Although the fillets had a very slight cinnamon odor after being treated with CA, the concentration of 0.2% (w/v) did not cause unfavorable impact on the original flavor of fillets in our preliminary experiment, which was similar to the findings reported by Zhang *et al.*^7^ and Hu *et al.*^45^ Sensory results of flounder fillets were tested at 4 day intervals during the entire storage period. As described in Fig. 6, as the storage time increased, a continuous decrease in the sensory scores was observed in all samples, indicating that the odor of flounder fillets gradually changed from pleasant to rancid with the extension of storage time. After 12 days of storage, the score of the control sample approached 3.9 exceeding the threshold of sensory rejection.^46^ However, the sensory evaluations of treated samples were notably better than those of the control sample, approaching the scores of 6.1, 6.2 and 6.9 for the UP, CA, and UP + CA groups, respectively. As storage time went on, UP and/or CA treatment gradually had a positive impact on the sensory quality of fillets, especially at the end of storage. This was in accordance with the results of TMA and ATP-related compounds. Sensory deterioration was attributed to bacteria spoilage and oxidation reactions, eventually resulting in the production of off-odor components, including volatile aldehydes and TMA.^47^ Compared with the control, higher scores were obtained in the treated samples, suggesting that UP and/or CA treatment was effective in slowing flounder fillets' sensory deterioration and preserving their flavor quality, particularly with UP + CA treatment. High pressure treatment could inhibit the formation of biogenic amines and improve the odor and taste of fish flesh, leading to better sensory quality compared with the untreated sample during storage.^16^ Moreover, Zhang *et al.*^7^ reported that vacuum-packaged common carp treated with CEO had better sensory quality than the control samples during refrigerated storage.

## 4. Conclusions

In summary, the present study indicated that a combination of CA and UP treatment had more positive effects on increasing IMP and umami-related amino acids, and reducing off-flavor nucleotides and bitter amino acids in refrigerated flounder fillets than did CA or UP treatments by themselves. In addition, the combination of CA and UP was more effective in reducing TVC, TCA-soluble peptides and putrid compound TMA. Combining the results of E-nose analysis and sensory evaluation, it can be concluded that CA combined with UP treatment might be a promising method to retain flavor quality of fish fillet and improve its edible quality during refrigerated storage.

## Conflicts of interest

The authors declared that there are no conflicts of interest associated with this work.

## Supplementary Material
